# Post-COVID-19 syndrome risk factors and further use of health services in East England

**DOI:** 10.1371/journal.pgph.0001188

**Published:** 2022-11-30

**Authors:** Maciej Debski, Vasiliki Tsampasian, Shawn Haney, Katy Blakely, Samantha Weston, Eleana Ntatsaki, Mark Lim, Susan Madden, Aris Perperoglou, Vassilios S. Vassiliou

**Affiliations:** 1 Norwich Medical School, University of East Anglia, Norwich, United Kingdom; 2 Cardiology Department, Norfolk and Norwich University Hospitals NHS Foundation Trust, Norwich, United Kingdom; 3 Norfolk and Waveney Integrated Care Board, Norwich, United Kingdom; 4 Rheumatology Department, East Suffolk and North Essex Foundation NHS Trust, Ipswich Hospital, Ipswich, United Kingdom; 5 Centre for Rheumatology, University College London, London, United Kingdom; 6 School of Mathematics, Statistics and Physics, Newcastle University, Newcastle upon Tyne, United Kingdom; 7 Institute of Continuing Education, University of Cambridge, Cambridge, United Kingdom; Malawi Liverpool Wellcome Clinical Research Programme / Kamuzu University of Health Sciences, MALAWI

## Abstract

Post-COVID syndrome, defined as symptoms persisting for more than twelve weeks after the diagnosis of COVID-19, has been recognised as a new clinical entity in the context of SARS-CoV-2 infection. This study was conducted to characterise the burden and predictors for post-COVID-19 syndrome in the local population. It was a community-based web-survey study conducted in Norfolk, East England, UK. We sent the survey to patients with confirmed COVID-19 infection by real-time polymerase chain reaction by December 6th, 2020. Questions related to the pre-COVID and post-COVID level of symptoms and further healthcare use. Baseline characteristics were collected from the primary care records. Logistic regression analysis was conducted to establish predictors for post-COVID-19 syndrome and further healthcare utilisation. Of 6,318 patients, survey responses were obtained from 1,487 participants (23.5%). Post-COVID-19 syndrome symptoms were experienced by 774 (52.1%) respondents. Male sex compared to female sex was a factor protective of post-COVID symptoms; relative risk (RR) 0.748, 95% confidence interval (CI), 0.605–0.924. Body mass index was associated with a greater risk of developing post-COVID-19 symptoms (RR 1.031, 95% CI, 1.016–1.047, for 1 kg/m2). A total of 378 (25.4%) people used further health services after their index COVID-19 infection, of whom 277 (73.2%) had post-COVID symptoms. Male sex was negatively associated with the use of further health services (RR 0.618, 95% CI, 0.464–0.818) whereas BMI was positively associated (RR 1.027, 95% CI, 1.009–1.046). Overall, post-COVID-19 symptoms increased the probability of using health services with RR 3.280, 95% CI, 2.540–4.262. This survey of a large number of people previously diagnosed with COVID-19 across East England shows a high prevalence of self-reported post-COVID-19 syndrome. Female sex and BMI were associated with an increased risk of post-COVID-19 syndrome and further utilisation of healthcare.

## Introduction

Post-COVID-19 syndrome, also referred to as Long COVID syndrome, is a term used to describe a condition faced by patients presenting with signs and symptoms that develop during or after infection with COVID-19, continue for more than 12 weeks and are not explained by an alternative diagnosis. When signs and symptoms of COVID-19 only persist from 4 to 12 weeks, the National Institute for Health and Care Excellence (NICE) defined it as ongoing symptomatic COVID-19, whilst when exceeding 12 weeks this was defined as Post-COVID-19 syndrome [1]. The complex condition affects people in different ways, with breathlessness, cough, heart palpitations, headaches, and severe fatigue being among the most prevalent symptoms. However, the symptoms commonly include also chest pain or tightness, problems with memory and concentration ("brain fog"), difficulty sleeping (insomnia), dizziness, pins and needles, joint pain, depression and anxiety, tinnitus, earaches, nausea, diarrhoea, stomach aches, loss of appetite, a high temperature, headaches, sore throat, changes to sense of smell or taste and rashes [1]. This is a new condition and due to the lack of validated tools to effectively assess and manage, primary care and hospital outpatient clinics are being overwhelmed due to the sheer numbers of patients seeking support [1,2].

Given the global scale of this pandemic, there has been a rapid effort to characterise the burden and find predictors for post-COVID-19 syndrome. As of March 24th 2022, the World Health Organisation estimates there have been 472,816,657 confirmed cases of COVID-19, including 6,099,380 deaths worldwide, whereas in the UK, there have been 20.75 million people testing positive and 164.734 deaths [3,4]. The Office for National Statistics, UK (ONS) estimates that as of 2 January 2022, 1.3 million people living in private households in the UK (2.1% of the population) were experiencing self-reported symptoms persisting for more than four weeks after the first suspected coronavirus (COVID-19) infection that were not explained by something else [5]. Ongoing symptomatic COVID-19 or post-COVID-19 syndrome is estimated to be adversely affecting the day-to-day activities of 836,000 people in the United Kingdom according to the ONS report with 244,000 saying their ability to undertake day-to-day activities had been "limited a lot".

There is growing evidence that having the vaccine reduces the risk of developing post-COVID-19 syndrome [6]. However, the disease mechanisms causing post-COVID-19 syndrome are unknown, and there are no evidence-based treatment options to alleviate the symptoms or reduce the duration [7].

This study was conducted to characterise the burden and predictors for post-COVID-19 syndrome in Norfolk, East England, United Kingdom and to build a statistical model that can inform regional post-COVID-19 syndrome healthcare service planning.

## Methods

### Study design

This study was a community-based cross-sectional study conducted in Norfolk, East England, UK as a part of Protect Norfolk and Waveney post-COVID-19 syndrome Project (NoW). The project was established to understand the prevalence and ongoing needs of the local population, who are experiencing the longer-term effects of post-COVID-19 syndrome. A web-based survey was developed based on: COVID-19 Yorkshire Rehab Screening Tool (C19-YRS) [8], COVID-19 rapid guideline: managing the long-term effects of COVID-19 (NG188) [1], and Medical Research Council (MRC) Dyspnoea Scale [9]. C19-YRS questionnaire is a clinically validated outcome measure tool recommended by the National Health Service (NHS) England and the National Institute for Health and Care Excellence (NICE) to routinely capture the severity of symptoms that persist longer than four or more weeks after contracting COVID-19 [1,10].

Patients were sent an invitation in February 2021 via a letter to complete the online survey consisting of a mixture of 39 single-choice yes/no or Likert scale questions (Supplementary Material). Questions related to pre-COVID and post-COVID breathlessness, use of any health services in relation to post-COVID-19 syndrome, chest pain, loss of sense of taste or smell, difficulty eating or drinking, weight loss or nutritional concerns, problems with walking, fatigue, short-term memory problems, new bowel concerns, any pain, severity of current anxiety and depression compared to pre-COVID levels, presence of dreams related to COVID illness or hospital admission, problems with communication. Patients were deemed to suffer from post-COVID-19 syndrome when they selected one or more new self-reported symptoms from the questionnaire. Multiple participation was not possible. The survey design did not require the individuals to answer all questions. Approval for this work was granted by the Control Of Patient Information (COPI) notice, approved by the Secretary of State for Health and Social Care for COVID19 research, and Institutional Approval received by the NHS Norfolk and Waveney Integrated Care Board. Participants received an invite by post which included consent information explaining the purpose of the survey. The consent was implied when participants logged in and provided their responses. The anonymity of participants was ensured by full anonymisation before data extraction.

The patient-identifiable data was processed under the control of patient information (COPI) notice. COPI was changed in March 2020 under the COVID-19 Public Health Directions to support various Covid-19 purposes. Under those regulations patient information was to be shared and used for the purposes of informing health services planning and supporting vital research on the cause, effects, treatment and outcomes for patients with the virus. Protect NoW Project was approved and signed off with our Population Health Clinical Team. We reported the results in accordance with the Checklist for Reporting Of Survey Studies (CROSS) [11].

### Patient and public involvement

No patient and public involvement group input was obtained for this study. However, follow-up letters have been issued to signpost participants to further support.

### Participants

Study inclusion criteria were: confirmed COVID-19 infection by real-time polymerase chain reaction (PCR) between the start of COVID-19 pandemic and December 6^th^ 2020. Exclusion criteria included residents of care or nursing homes and South Norfolk residence.

### Variables

Demographics, comorbidities, medications, smoking status and deprivation index were collected from clinical National Health Service Digital databases. Deprivation was expressed as a deprivation decile and was measured using the Index of Multiple Deprivation (IMD), which provides an overall relative measure of deprivation for each lower layer super output area (LSOA) [12]. An LSOA is a small area with an average population of 1,500 people. The overall IMD scores are ranked for all LSOAs within a country and can be divided into 10 groups (deciles) where decile 1 represents the most deprived LSOAs and decile 10 represents the least deprived LSOAs. The IMD is a score based on the area as a whole and not everyone within an LSOA necessarily experiences the same level or type of deprivation. Body mass index (BMI) was calculated based on the most current General Practice records. Medications were categorised into the following categories: beta-blockers, calcium channel blockers, diuretics, oral steroids, angiotensin-converting enzyme inhibitors (ACEI) or angiotensin receptor blocker (ARB), antiplatelets, statins, antidepressants, insulin, oral diabetic medications, anticoagulants, proton pump inhibitors. Previous administration of COVID-19 vaccine was collected from NHS records.

#### Statistical analysis

The data were analysed by an independent professional statistician who had access to all data underpinning this study. For all analyses, a 2-sided p<0.05 was considered statistically significant. All data were processed using R statistical package. Standard descriptive statistics methods were used to describe data, means with standard deviations (SD) for continuous variables and frequency tables for categorical. Missing values were reported for BMI (608 out of 1487) and deprivation index (114 out of 1487). For those we used multiple imputation with 30 iterations in MICE (R). We used a multivariable logistic regression model to predict the probability of post-COVID-19 syndrome. A full model with all covariates was assessed. We also used backward variable selection to identify the most important variables in the model. Risk profiles were based on the model with variable selection.

## Results

Of 6,318 patients, survey responses were obtained from 1,487 participants (23.5%). Females accounted for 61% of the cohort (n = 907). Mean age of participants was 50, SD 18.1. BMI was available for 879 participants, mean 28.4, SD 6.9 kg/m^2^. Current smoking declared 81 participants, 395 were ex-smokers, 704 never smoked and information was not available for 307 participants. Mean deprivation decile was 5.3, SD 2.4. Comorbidities and baseline medications are presented in Tables 1 and 2. Eleven people had received their first dose of COVID mRNA Vaccine BNT162b2.

**Table 1:** Baseline comorbidities.

*Comorbidities*	Number of participants (n = 1,487)
ADHD	6 (0.4%)
Angina	28 (1.9%)
Anxiety	202 (13.6%)
Arthritis	18 (1.2%)
Asthma	195 (13.1%)
Atrial Fibrillation	47 (3.2%)
Bipolar Affective Disorder	6 (0.4%)
Bowel Cancer	4 (0.3%)
Breast Cancer	18 (1.2%)
CDND Cerebral Palsy MS	76 (5.1%)
Chronic Heart Disease	72 (4.8%)
Chronic Kidney Disease	29 (2.0%)
Chronic Liver Disease	7 (0.5%)
Chronic Obstructive Pulmonary Disease	36 (2.4%)
Chronic Respiratory Disease	71 (4.8%)
Coronary Heart Disease	57 (3.8%)
Dementia	8 (0.5%)
Depression	242 (16.3%)
Diabetes Type 1	12 (0.8%)
Diabetes Type 2	87 (5.9%)
Eating Disorder	24 (1.6%)
Heart Failure	17 (1.1%)
Hypertension	224 (15.1%)
Immunosuppression	18 (1.2%)
Inflammatory Bowel Disease	84 (5.6%)
Irritable Bowel Syndrome	107 (7.2%)
Male Genito-Urinary Cancer	17 (1.1%)
Moderate Frailty	21 (1.4%)
Myocardial Infarction	17 (1.1%)
NULL	642 (43.2%)
Other Cancer	22 (1.5%)
Palliative Care	9 (0.6%)
Peripheral Vascular Disease	9 (0.6%)
Psychosis	4 (0.3%)
Pulmonary Embolism	19 (1.3%)
Retinopathy	22 (1.5%)
Rheumatoid Arthritis	15 (1.0%)
Rheumatology	30 (2.0%)
Self-Harm	11 (0.7%)
Severe Frailty	12 (0.8%)
Skin Cancer	29 (2.0%)
Stroke Transient Ischaemic Attack	56 (3.8%)
Supportive Care Register	5 (0.3%)
Vascular Disease	48 (3.2%)

**Table 2:** Medications at the time of study participation.

*Medications*	Number of participants (n = 1,487)
ACEI/ARB	44 (3.0%)
Anticoagulation	15 (1.0%)
Antidepressants	44 (3.0%)
Antiplatelets	25 (1.7%)
Calcium channel blockers	19 (1.3%)
Beta-blockers	26 (1.7%)
Diuretics	16 (1.1%)
Insulin	4 (0.2%)
Oral diabetic medications	19 (1.3%)
Proton pump inhibitors	61 (4.1%)
Statins	52 (3.5%)
Oral steroids	11 (0.7%)

There were 774 (52.1%) respondents who had experienced post-COVID-19 syndrome symptoms. Incidence of post-COVID-19 syndrome symptoms in women was 55.9% and in men 46.0% (p<0.001).

**Fig 1:**
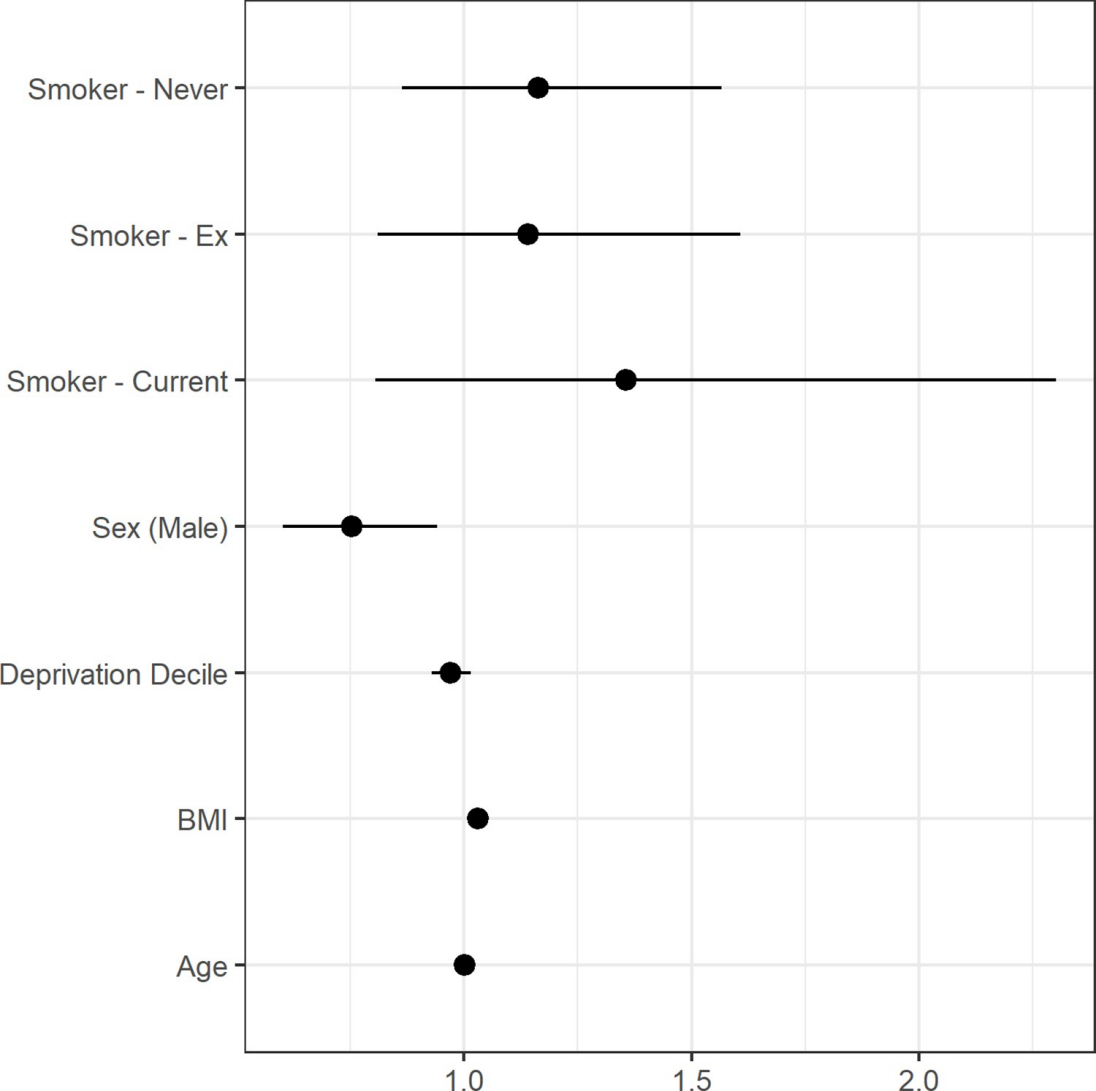
Multivariable regression model analyzing potential risk factors for post-COVID-19 syndrome.

**Fig 2:**
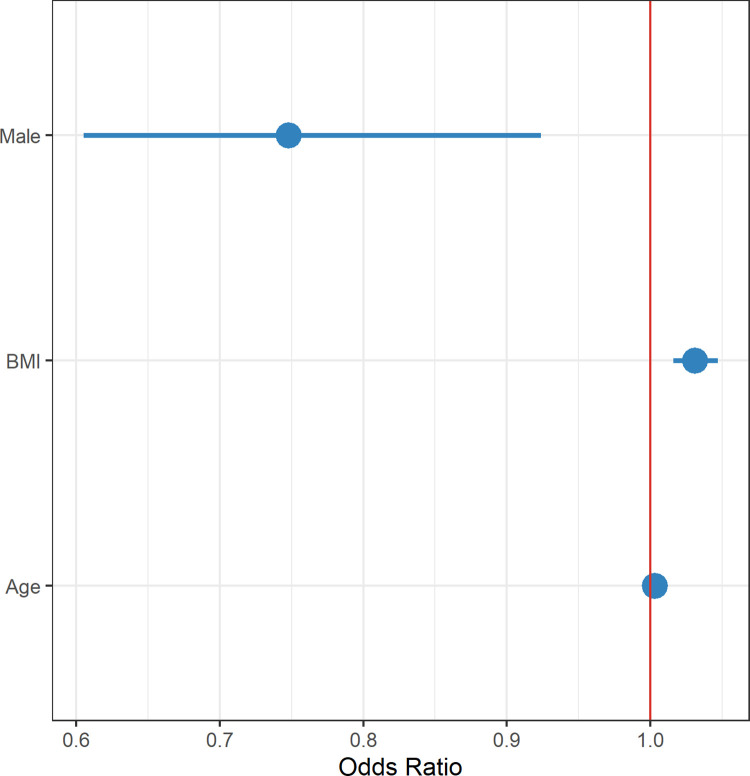
Regression model for covariates with p<0.1. Sex and BMI were statistically significant whilst age showed a trend towards significance.

In regression analyses (Figs 1 and 2), male sex compared to female sex was a factor protective of post-COVID symptoms; relative risk (RR) 0.748, 95% confidence interval (CI), 0.605–0.924. Body mass index was associated with greater risk of developing post-COVID19 symptoms, with patients having 1.031 times higher chance of suffering with these symptoms for each 1 kg/m^2^ increase their BMI (RR 1.031, 95% CI, 1.016–1.047). Age per 1-year increase was not found to be an independent risk factor for higher incidence of post-COVID19 symptoms (RR 1.003, 95% CI, 0.998–1.009). Based on the above, it can be estimated that for a man to have the same probability as female of same age with normal BMI they have to have a BMI over 35 kg/m^2^.

**Fig 3:**
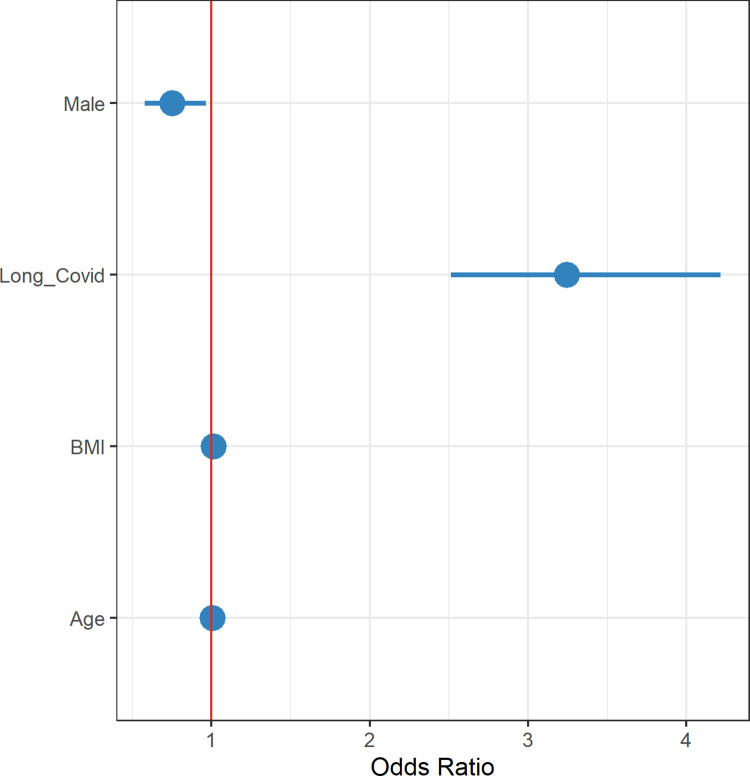
Analysis of 378 patients that used further health services. Suffering from Post-COVID-19 syndrome was the leading factor requiring any further health services input.

**Fig 4:**
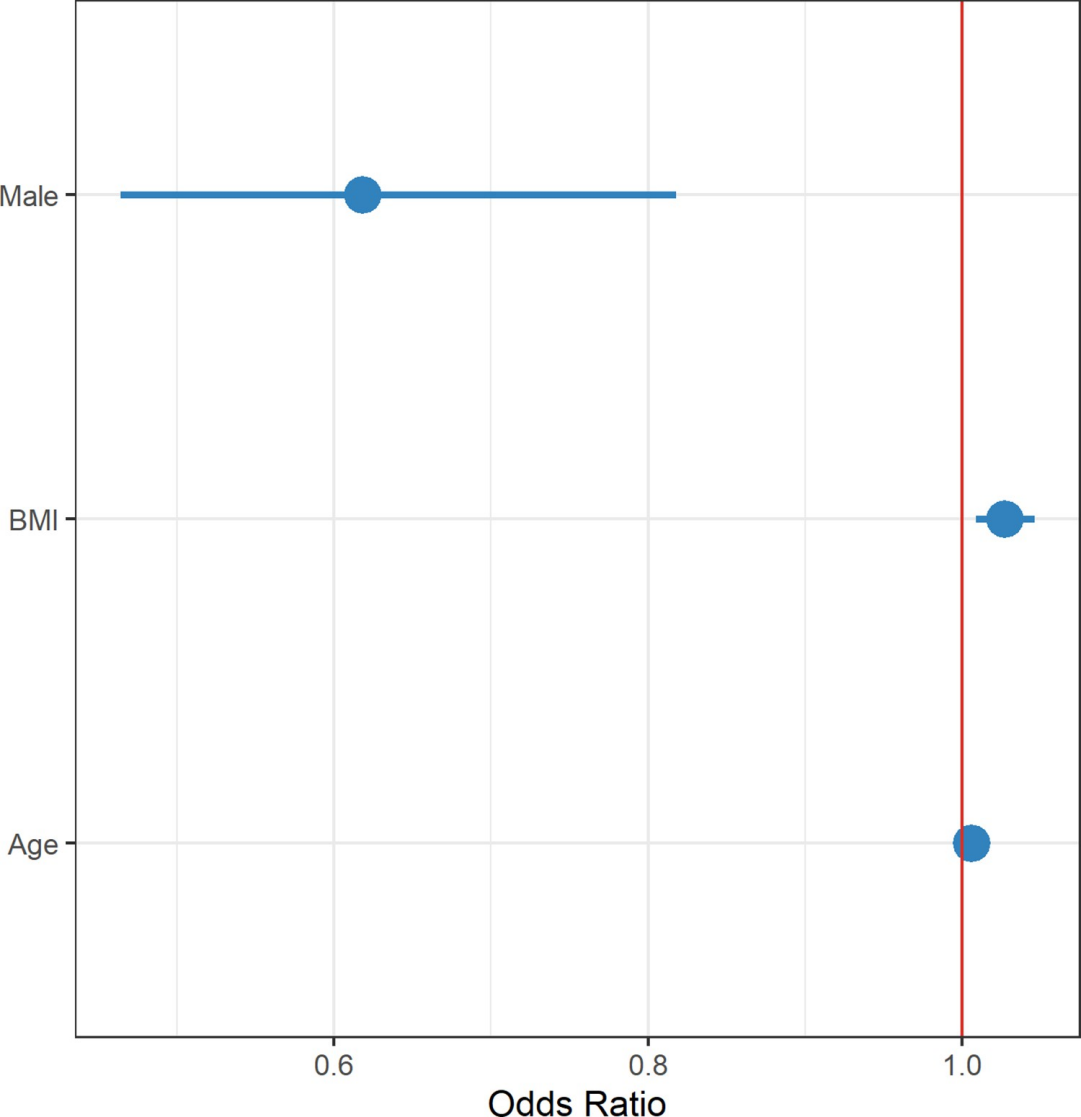
Predictors of using health services when having post-COVID-19 syndrome symptoms (analysis for 277 patients with post-COVID-19 syndrome and further use of health services). Higher BMI associated with increased use, whilst being a man associated with less use of services.

A total of 378 people used further health services after their index COVID-19 infection, of whom 277 (73.2%) had post-COVID symptoms. Patients suffering with post-COVID-19 syndrome were significantly more likely to seek further help and advice from health services compared to those who did not have post-COVID-19 syndrome (RR 3.28, 95% CI, 2.54–4.26). Additionally, it was noted that men were much less likely than women to use further health services (RR 0.750, 95% CI, 0.580–0.966) (Fig 3). In keeping with this, in a further detailed analysis of 277 patients with post-COVID19 syndrome, it was demonstrated that only 14.5% (84/580) of men used further health services compared to 21.3% (193/907) of women. Regression analysis showed that male sex was negatively associated with the use of further health services, (RR 0.618, 95% CI, 0.464–0.818) while BMI was a positive predictive indicator of this (RR 1.027 for each 1 kg/m^2^ increase, 95% CI 1.009–1.046) (Fig 4). Overall, in the whole population, post-COVID-19 symptoms increased the probability of using health services with RR 3.280, 95% CI 2.540–4.262.

## Discussion

The results of this study provide a snapshot of self-reported symptoms after recent confirmed COVID-19 infection in the East of England. It was found that female sex and high BMI are associated with higher likelihood of developing post-COVID19 syndrome. Those two factors have a significant predictive value in the use of further health services among those diagnosed with post-COVID19 syndrome.

The reported incidence of post-COVID-19 syndrome in the present study amounted to 52% and was concordant with a meta-analysis based on studies published before January 2021 showing that approximately half of the individuals (53%, 95% CI: 41–65%) reported persistence or presence of one or more symptoms over 12 weeks after COVID-19 diagnosis [13]. The study notes that the most prevalent symptoms were: anxiety (32%), general pain or discomfort (28%), fatigue (25%), insomnia (22%) and cognitive impairment (20%). Another meta-analysis of studies published before 1^st^ January 2021 showed that 80% (95% CI 65–92) of the patients with COVID-19 have long-term symptoms [14]. In that study, the 5 most common manifestations were fatigue (58%), headache (44%), attention disorder (27%), hair loss (25%), dyspnoea (24%).

A study conducted on data of all people registered with a primary care general practice (GP) in November 2020 encompassing 96% of the English population between 1 February 2020 and 25 April 2021 assessed the use of post-COVID-19 syndrome codes in GP Practices [15]. Up to 25 April 2021, there were 23,273 (0.04%) patients with a recorded code indicative of a post-COVID-19 diagnosis. Interestingly, the rate of documented post-COVID19 diagnosis varied substantially between regions, from a minimum of 20.3 per 100 000 people in the East of England (95% CI,19.3–21.4) to a maximum of 55.6 in London (95% CI, 54.1–57.1). To date, this study provides the most comprehensive insights into the post-COVID-19 syndrome prevalence and risk factors in a demographically distinct population of East of England–one of the eldest populations in the UK [15,16].

Our study showed that female sex and high BMI are important predictive factors for the development of post-COVID19 syndrome. This is in keeping with a previous study conducted in England, which showed that females had a higher rate of recorded post-COVID-19 syndrome codes than males (52.1 versus 28.1 per 100,000 people) [15]. According to ONS, the prevalence of any post-COVID-19 syndrome symptoms is higher in women compared with men (23.6% versus 20.7%), while the age group estimated to be most significantly affected by post-COVID-19 syndrome symptoms is 35–49 years (26.8%), followed by 50–69 years (26.1%), and the ≥70 years group (18%) [5]. Obesity has been associated with an increased need for diagnostic tests starting from 30 days after a positive SARS-CoV-2 test with a hazard ratio of 1.39 (95% CI 1.13 to 1.71) for severe obesity (BMI≥40) versus normal BMI (18–24 kg/m^2^), and an HR of 1.25 (95% CI 1.02 to 1.53) for moderate obesity (BMI 35–39) versus normal BMI [17]. The authors noted that there was a higher rate of cardiac, vascular, pulmonary, gastrointestinal, and mental health related testing among obese patients than those with normal BMI.

A Coronavirus (COVID-19) Infection Survey (CIS) performed by ONS in the UK, showed that among participants with COVID-19 who were exactly one-to-one matched to control participants on a number of factors, 5.0% out of 12,611 participants reported symptoms at 12 to 16 weeks which was statistically significantly higher than in the control group (3.4%) [18]. The difference in prevalence remained statistically significant at 20 to 24 weeks. The results of the CIS study suggest that the prevalence of symptoms comprising fever, headache, muscle ache, weakness/tiredness, nausea/vomiting, abdominal pain, diarrhoea, sore throat, cough, shortness of breath, loss of taste, and loss of smell following COVID-19 infection is greater than the background prevalence of these symptoms in the population.

As a response to challenges posed by the post-COVID-19 syndrome on healthcare needs and patients’ ability to work along with lack of established treatments for people living with post-COVID-19 syndrome several trials has been launched in the UK looking at improving home monitoring and self-management of symptoms and identifying effective treatments [19]. ReDIRECT trial is testing the hypothesis of whether a well-established weight management programme, delivered and supported remotely, can improve symptoms for people with post-COVID-19 syndrome and overweight/obesity [20]. STIMULATE-ICP, the largest long COVID trial to date, recruiting more than 4,500 people with the condition, will test the effectiveness of existing drugs to treat post-COVID-19 syndrome and the impact on patients’ symptoms, mental health and outcomes such as returning to work [21].

### Limitations

The survey responses were received around three months after the first Covid vaccination was administered in the UK on the 8^th^ of December 2020. By the time of the first response, many people received their first dose COVID-19 vaccine in the UK. Therefore, this survey represents the post-COVID-19 syndrome picture in the largely pre-vaccination era and the findings cannot be generalised to populations with a high percentage of vaccinated people. Also, as the new SARS-CoV-2 have emerged, the results of this study relate to pre-Omicron variants. Having said this, whilst the vaccines have reduced the number of individuals suffering from post-COVID-19 syndrome, it is unlikely that they would have altered the risk factors for this. Likewise, the new variants are unlikely to have changed the risk factors either.

The response rate was 23.5% which although appropriate for a survey, could raise the possibility of non-response bias if the responders differed from the non-responder in terms of baseline characteristics and prevalence of post-COVID-19 syndrome symptoms which could not be evaluated in this study. The selection bias was minimised as the survey was sent to all patients with confirmed COVID-19 diagnosis in the regions of interest. As our study involved self-reporting symptoms and use of further health services by participants there is a considerable risk of response bias. We did not have a SARS-CoV-2 negative control group and did not explore if other conditions could explain the reported symptoms. In addition, we did not report the meantime from COVID-19 diagnosis to survey completion which may have contributed to the underestimation of the reported prevalence of post-COVID-19 syndrome.

## Conclusion

The present survey of a large number of people previously diagnosed with COVID-19 across East of England shows a high prevalence of post-COVID-19 syndrome. The results suggest females and increased BMI were associated with an increased risk of post-COVID-19 syndrome and further utilisation of health care, with increasing age showing a trend towards significance. The survey results provide valuable insights to help plan the local, national and international integrated referral pathway and assessment centres.

## Supporting information

S1 File Survey questions. (DOCX)
