# Supplementary material for: Post-COVID-19 syndrome risk factors and further use of health services in East England
Source: PLOS Glob Public Health. 2022 Nov 30;2(11):e0001188. doi: 10.1371/journal.pgph.0001188 (PMC10022108; doi:10.1371/journal.pgph.0001188)
Supplement: S1 File — (DOCX) [file pgph.0001188.s001.docx]

S1 File: Survey questions.

| Question number | Question | Answer type |
| --- | --- | --- |
| 1 | Were you admitted to hospital because of Covid-19? | Yes No |
| 2 | Are you still troubled by symptoms of COVID-19?  If answering no, please skip the remaining questions and click save and submit at the bottom of the questionnaire. | Yes No |
| 3 | Have you had any further medical problems or needed to go to hospital since your experience with Covid-19? | Yes No |
| 4 | Have you used any other health services since your experience with Covid-19? (e.g. your GP) | Yes No |
| 5 | Please rate your breathlessness after your experience with Covid-19. | Options |
| 6 | Please rate your breathlessness pre-Covid-19 | Options |
| 7 | Have you been experiencing any chest pain? | Yes No |
| 8 | Do you get any palpitations? (the sense that you can feel your heart pounding or racing) | Yes No |
| 9 | Have you lost your sense of taste? | Yes No |
| 10 | Have you lost your sense of smell? | Yes No |
| 11 | Are you having difficulty eating, drinking or swallowing such as coughing, choking or avoiding any food or drinks? | Yes No |
| 12 | If you are having difficulties swallowing, please rate the impact. | Scale |
| 13 | Are you or your family concerned that you have ongoing weight loss or any ongoing nutritional concerns as a result of COVID-19? | Yes No |
| 14 | Please rank your appetite or interest in eating. | Scale |
| 15 | How severe are any problems you have in walking about? (or moving about if normally mobilises in another way) | Scale |
| 16 | How severe were any problems you had in walking about before you had COVID (or moving about if normally mobilises in another way) | Scale |
| 17 | Do you become fatigued more easily compared to before your illness? | Yes No |
| 18 | If you are becoming more fatigued since your illness, how severely does this affect your mobility, personal care, activities and enjoyment in life? | Scale |
| 19 | How severely did becoming fatigued pre-COVID affect your mobility, personal care, activities and enjoyment in life? | Scale |
| 20 | How severe are any problems you have in personal cares such as washing and dressing yourself? | Scale |
| 21 | How severe were any problems you may have had pre-COVID in personal cares such as washing and dressing yourself? | Scale |
| 22 | Since your illness, are you having any NEW problems with controlling your bowels? | Yes No |
| 23 | Since your illness, are you having an NEW problems with controlling your bladder? | Yes No |
| 24 | How severe are any problems you have in doing your usual activities, such as your household role, leisure activities, work or study? | Scale |
| 25 | How severe were any problems pre-COVID you may have had in doing your usual activities, such as your household role, leisure activities, work or study? | Scale |
| 26 | How severe is any pain or discomfort you have? | Scale |
| 27 | How severe is any pain or discomfort you had pre-COVID? | Scale |
| 28 | Since your illness, have you had any new or worsened difficulty with concentrating? | Yes No |
| 29 | Since your illness, have you had any new or worsened difficulty with short term memory? | Yes No |
| 30 | Have you or your family noticed any change in the way you communicate with people, such as making sense of things people say to you, putting thoughts into words, difficulty reading or having a conversation? | Yes No |
| 31 | If there has been a difference in communication, please rate the impact | Scale |
| 32 | How severe is any anxiety you may be experiencing? | Scale |
| 33 | How severe was any anxiety pre-COVID? | Scale |
| 34 | How severe is any depression you may be experiencing? | Scale |
| 35 | How severe was any depression you may have had pre-COVID? | Scale |
| 36 | Have you had any unwanted memories of your illness or hospital admission whilst you were awake, not counting dreams? | Yes No |
| 37 | How good or bad is your health overall? | Scale |
| 38 | How good or bad was your health pre-COVID? | Scale |
| 39 | Would you be happy to be contacted by a NHS healthcare professional? | Yes No |
